# Interaction does Count: A Cross-Fostering Study on Transgenerational Effects of Pre-reproductive Maternal Enrichment

**DOI:** 10.3389/fnbeh.2015.00320

**Published:** 2015-12-01

**Authors:** Paola Caporali, Debora Cutuli, Francesca Gelfo, Daniela Laricchiuta, Francesca Foti, Paola De Bartolo, Francesco Angelucci, Laura Petrosini

**Affiliations:** ^1^Department of Psychology, Faculty of Medicine and Psychology, University “Sapienza” of RomeRome, Italy; ^2^I.R.C.C.S., Santa Lucia FoundationRome, Italy; ^3^Department of Systemic Medicine, University of Rome “Tor Vergata”Rome, Italy; ^4^Department of Sociological and Psychopedagogical Studies, University “Guglielmo Marconi” of RomeRome, Italy

**Keywords:** environmental enrichment, cross-fostering, maternal care, cognition, BDNF, rats

## Abstract

Pre-reproductive environmental enrichment of female rats influences sensorimotor development and spatial behavior of the offspring, possibly through the changed maternal nurturing. Nevertheless, maternal care could be not the solely responsible for changing offspring developmental trajectories. To disentangle the specific contribution to the transgenerational inheritance of pre- and post-natal factors, a cross-fostering study was performed. Female rats were reared in an enriched environment from weaning to sexual maturity, while control female rats were reared under standard conditions. Following mating with standard-reared males, all females were housed individually. Immediately after delivery, in- or cross-fostering manipulations were performed so that any foster dams received pups born to another dam of the same (in-fostering) or the opposite (cross-fostering) pre-reproductive rearing condition. In lactating dams maternal care and nesting activities were assessed, while in their male pups spatial abilities were assessed through Morris Water Maze (MWM) test at post-natal day 45. Moreover, the expression of Brain-Derived-Neurotrophic-Factor (BDNF) was evaluated in the hippocampus and frontal cortex of dams and pups at weaning. Pre-reproductive maternal environmental enrichment, followed by adoption procedures, loosened its potential in modifying maternal care and offspring developmental trajectories, as indicated by the lack of differences between in-fostered groups of dams and pups. In addition, enriched dams rearing standard pups showed the least complex maternal repertoire (the highest sniffing duration and the lowest nest quality), and their pups showed a reduced spatial learning in the MWM. Nevertheless, pre-reproductive maternal enrichment kept influencing neurotrophic pattern, with enriched dams expressing increased frontal BDNF levels (regardless of the kind of fostered pups), and their offspring expressing increased hippocampal BDNF levels. The present findings enlighten the crucial importance of the early mother-pups interactions in influencing maternal care and offspring phenotype, with the enriched dam-standard pups couple resulting in the most maladaptive encounter. Our study thus sustains that the bidirectional interactions between mother and pups are able to deeply shape offspring phenotype.

## Introduction

The transfer of phenotypic traits acquired by parents to the offspring is a debated process in biology since its promotion by Lamarck (1809). In fact, for a long time the contribution of nature and nurture in shaping phenotype has been considered as opposite. However, a growing body of evidence has recently revealed that the environmental experiences could be imprinted on the genome through epigenetic mechanisms, which influence DNA function without altering DNA sequence (Franklin and Mansuy, 2010; Ho and Burggren, 2010). Interestingly, the epigenetic phenomenon that imprints parental environmental experiences on the offspring genome can lead to different phenotypes that can persist over generations (Weaver, 2007).

The *environmental enrichment* (EE), originally defined by Rosenzweig et al. (1964) as “a combination of complex inanimate and social stimulations”, is a widely used paradigm to investigate the influence of complex sensorimotor, cognitive, and social stimulations on brain and behavior (Nithianantharajah and Hannan, 2009). Although the current literature suggests complex interactions among the time window, type of enrichment, and gender of enriched animals (Girbovan and Plamondon, 2013), the majority of studies reported beneficial effects on behavior (improved motor and cognitive abilities), brain and neuronal morphology (increased brain weight, neurogenesis, dendritic arborization, spines, and synaptic density) and molecular biology (changes in gene expression, modulation of neurotrophin, and neurotransmitter systems) following EE exposure (Nithianantharajah and Hannan, 2006; Petrosini et al., 2009; Baroncelli et al., 2010; Simpson and Kelly, 2011; Sale et al., 2014). Anyway, scattered negative outcomes have also been reported, probably linked to the enhanced stress levels induced by the EE protocol (Schilling et al., 2004; Wood et al., 2011; Huzard et al., 2015; Mo et al., 2015).

Interestingly, the most enduring EE effects have been described when the complex housing started immediately after weaning; not by chance, the first month of life is a critical and very sensitive time window, during which experience strongly modulates the development (Magalhaes et al., 2007). In the last years, increasing attention has been paid to the transgenerationally transmitted beneficial effects of parental EE exposure (Arai et al., 2009; Leshem and Schulkin, 2012; Mashoodh et al., 2012; Mychasiuk et al., 2012; Caporali et al., 2014; Cutuli et al., 2015). Overall, these reports show that the exposure of the parent to an EE has the potential to prepare the fetus to cope with a specific environment, promoting offspring fitness and influencing their cognitive behavior. Unfortunately, these studies did not examine the issue of maternal care (Arai et al., 2009; Leshem and Schulkin, 2012; Mychasiuk et al., 2012), or described modifications of maternal care without providing information on maternal physiological changes and/or offspring cognitive abilities (Mashoodh et al., 2012). Thus, the existing reports on transgenerational effects largely neglected the possible impact that such conditions may have on the dam itself (Girbovan and Plamondon, 2013). Conversely, this issue deserves particular attention. The few indications present in literature demonstrate that in virgin females EE delays the expression of pup-oriented responses and increases anxiety without affecting stress physiological correlates (Mann and Gervais, 2011). Recently, we demonstrated that pre-reproductive EE of female rats positively influences maternal behaviors as well as sensorimotor development and spatial behavior of their offspring, and enhances the neurotrophin expression (Caporali et al., 2014; Cutuli et al., 2015). However, our previous studies did not allow distinguishing whether the effects of EE maternal exposure were transmitted to offspring before or after birth. In fact, the environment experienced by the pregnant mother may exert substantial effects on the intrauterine milieu and alter fetal development. During pregnancy, the maternal exposure to complex environments usually elicits positive effects on offspring neurodevelopment (Dell and Rose, 1987; Welberg et al., 2006; Leshem and Schulkin, 2012; Mychasiuk et al., 2012; Rosenfeld and Weller, 2012), even if also negative effects have been described (Cymerblit-Sabba et al., 2013). On the other hand, even maternal exposure to environmental toxins, drugs, or hormonal alterations (e.g., due to prenatal stress) has a deleterious impact on physical and behavioral offspring development (Rice and Barone, 2000; Mantovani and Calamandrei, 2001; Swanson et al., 2009; Thompson et al., 2009; Glover, 2011; Venerosi et al., 2012). Thus, transgenerational changes we previously demonstrated (Caporali et al., 2014; Cutuli et al., 2015) may have occurred through factors transmitted from mother to offspring via placenta or milk (Ho and Burggren, 2010). Furthermore, also the early mother-infant interaction during the first life stages could remarkably impact on offspring phenotype, as indicated by Meaney’s group studies (Weaver et al., 2004; Weaver, 2007; Champagne and Curley, 2009). Therefore, to determine whether transgenerational transmission from the pre-reproductively enriched mother to her offspring occurred before or after birth, a cross-fostering study was performed. This experimental procedure allows clarifying the specific weight of pre- and post-natal factors on epigenetic changes. To this aim, female rats were reared in an enriched environment from weaning to breeding. Females reared in standard conditions were used as controls. At 2.5 months of age all females were mated and then reared in standard conditions with their offspring, which thus never experienced the EE. At birth, cross- or in-fostering manipulations were performed so that pups born to an enriched or standard female were reared from a foster standard or enriched female. Maternal care and nesting activity were assessed in lactating dams, as we previously demonstrated that the pre-reproductive EE deeply influences maternal behavior (Cutuli et al., 2015). Spatial abilities of male pups were evaluated by means of Morris Water Maze (MWM) at post-natal day (pnd) 45, a time point demonstrated to reveal the maternal enrichment influence on offspring spatial learning (Cutuli et al., 2015). Furthermore, at weaning the expression of Brain Derived Neurotrophic Factor (BDNF), a neurotrophin specifically involved in synaptic plasticity, was evaluated in frontal and hippocampal areas of dams and offspring. This biochemical correlate was analyzed since we previously demonstrated that maternal EE increased BDNF expression in mothers and in pups (Cutuli et al., 2015).

## Materials and Methods

### Maternal Housing Conditions

At weaning (pnd 21), female Wistar rats were randomly assigned to enriched or standard rearing conditions. From pnd 21 to pnd 72, the Enriched Females (EF) were reared in groups of ten in a large cage (100 cm × 50 cm × 80 cm), containing wood shavings, a running wheel and colored plastic objects, following the enrichment protocol previously described (Petrosini et al., 2009; Cutuli et al., 2011, 2015; Foti et al., 2011; Caporali et al., 2014). On pnd 72, the EF were pair-housed in standard cages (40 cm × 26 cm × 18 cm) for a week to become accustomed to the standard cages before mating.

The Standard-reared Females (SF) were pair-housed in standard cages containing wood shavings and a red plastic tube. A 12/12 h dark/light cycle (light on between 07:00 and 19:00 h) was applied to both enriched and standard conditions. Food and water were provided *ad libitum*.

Before mating all females were weighted. For mating, from pnd 80 to pnd 85 (6 days), each EF and SF in oestrus stage was caged with a standard-reared male rat (≈300 g). Afterward, the males were removed, and the females were maintained in the standard home-cages throughout pregnancy, delivery and until offspring’s weaning (pnd 21).

All efforts were made to minimize animal suffering and reduce the number of animals that were used, per the European Directive (2010/63/EU). All procedures were approved by the Italian Ministry of Health.

### Experimental Groups of Dams and Pups

Within 6 h from delivery, mothers were removed from their home-cages and pups were sexed and counted. All litters were culled to 8 pups (five or six males and three or two females) and weighted. The six litters per group that were compliant with this condition were included in the present study. Twenty-four litters were in- or cross-fostered so that any foster dams received an entire culled litter born to another dam of the same (in-fostering) or the opposite (cross-fostering) pre-reproductive rearing condition (Bartolomucci et al., 2004). Each fostering procedure took less than 10 min.

Depending on the rearing conditions of the foster mother (**E** or **S**) and those of the biological mother (**e** or **s**), four groups of dams and four groups of male pups were obtained:

*Dams’ groups* (*n* = 6/group):

– **EeF** that encompassed EF that reared pups born to another EF (*in-fostering*);
– **SsF** that encompassed SF that reared pups born to another SF (*in-fostering*);
– **EsF** that encompassed EF that reared pups born to a SF (*cross-fostering*);
– **SeF** that encompassed SF that reared pups born to an EF (*cross-fostering*).

*Pups’ groups*:

– **EeP** that encompassed pups reared by an EF and born to another EF (*in-fostering*);
– **SsP** that encompassed pups reared by a SF and born to another SF (*in-fostering*);
– **EsP** that encompassed pups reared by an EF but born to a SF (*cross-fostering*);
– **SeP** that encompassed pups reared by a SF but born to an EF (*cross-fostering*).

Note that the difference in rearing conditions concerned the mothers in their pre-reproductive phase, but not the pups which were all reared in standard conditions.

**FIGURE 1:**
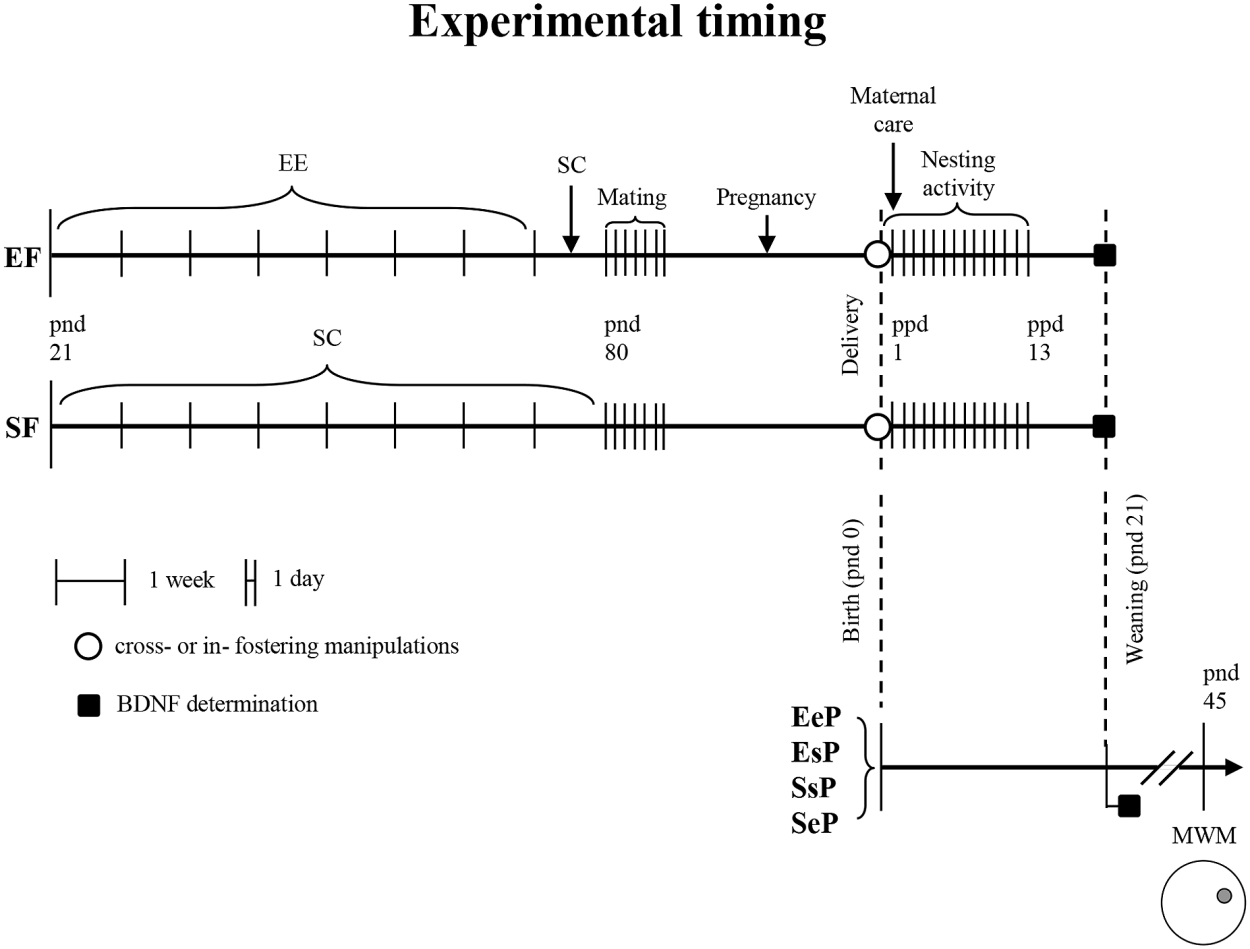
**Experimental timing: female rats reared according to different pre-reproductive conditions (EF: enriched females; SF: standard females); cross- or in- fostering procedures (○); behavioral testing (Nesting activity and Maternal care observations); biochemical analyses (■, BDNF determination).** Groups of male pups according to pre-reproductive conditions of foster and biological mothers (EeP: pups reared by an EF and born to another EF; EsP: pups reared by an EF but born to a SF; SsP: pups reared by a SF and born to another SF; SeP: pups reared by a SF but born to an EF); behavioral testing (MWM, Morris Water Maze). EE, environmental enrichment; SC, standard condition; pnd, post-natal day; ppd, post-partum day.

Maternal care and nesting activity were assessed in lactating dams. Twelve pups/groups (2–3 pups/litter) were submitted to MWM on pnd 45. Since endogenous BDNF release is activity-dependent (Kuczewski et al., 2009), to avoid eventual effects of behavioral testing on BDNF release, four pups/group (two pups/litter) not behaviorally tested were used to measure hippocampal BDNF levels at pnd 21. Frontal cortex BDNF determination was performed on dams at ppd 21 (*n* = 4/group). Male pups were weighted at weaning (*n* = 16/group) and at pnd 45 (*n* = 12/group). Global timing of experimental procedures is reported in **Figure 1** and the whole number of dams and pups used are reported in **Table 1**.

**Table 1:** Experimental groups of dams and pups.

	Behavioral analyses (*n*)	BDNF expression (*n*)	Total number
Dams	6/group = 24	4/group = 16 (out of 24 behaviorally tested dams)	24
Male pups	12/group = 48	4/group = 16 (not previously behaviorally tested)	48 + 16 = 64

### Behavioral Testing

#### Dams

##### Nest building activity

To test the nest building ability, daily from delivery day (ppd 0) to ppd 13 dams were given the opportunity to build a nest providing them 6 g of sterile surgical cotton placed on the lid of each cage (Cutuli et al., 2015). To analyze nest building propensity, the built nests were removed on ppd 1, 2, 4, 6, 9, 12. After nest removal new cotton was provided. Quantitative indices of nest building activity were the mean latency to manipulate cotton and weight of the used cotton. Nest building was considered to start when the female pulled down the cotton from the cage lid with forelimbs and mouth (cut-off time: 10 min).

The nest quality was assessed daily (ppd 1–13) by two independent observers blind to dams’ rearing conditions (inter-rate reliability > 0.9), using the following 4-point rating scale (Cutuli et al., 2015):

1 = no nest (the dam did not use at all cotton or sawdust to build the nest, or scattered cotton and sawdust throughout the home-cage with no clear nest shape);
2 = primitive flat nest (the dam used cotton to build a plane nest);
3 = complex cup-shaped nest (the dam used the cotton to build an open nest with walls);
4 = complex hooded nest (the dam built a round and well-shaped nest with walls forming a ceiling). Nesting quality total score obtained by summing the values from ppd 1 to 13 was analyzed.

##### Maternal care observations

In rats, maternal care consists of several behaviors toward the litter that ensure pups’ survival and promote offspring development. To obtain information about the effects of pre-reproductive maternal EE and cross-fostering on maternal care at early stages, mother-pups interactions were recorded on ppd 1 (Cutuli et al., 2015). The observations were made by a trained observer blind to the dams’ rearing conditions between 10 am and 4 pm.

Animals were habituated to the testing room for 10 min. Thirty minutes before the start of each observation session, the pups were removed (isolation period), the nest eliminated, and the dam remained alone in her home-cage. During the isolation period, the pups were weighted and then placed altogether in a small box at 32 ± 1°C. Notably, this brief maternal separation cannot be considered a maternal deprivation protocol (Vetulani, 2013), lasting less than 3 h. Conversely, given short periods of separation may stimulate maternal care (Liu et al., 1997), this procedure allowed studying maternal behavior in an eliciting condition and not in basal conditions.

At the beginning of each observation, the cage lid was gently replaced by a transparent perforated Plexiglas top, all pups were re-placed in their home-cage in the side opposite to the previously removed nest and dam’s behavior was video-recorded for 30 min. Duration of the following behaviors (Fleming and Rosenblatt, 1974; Petruzzi et al., 1995; Venerosi et al., 2008) was measured:

*- Pup-directed behaviors*:

– Retrieving: the dam was picking up any pup in her mouth and carrying it to the nest;
– Licking: the dam was licking or grooming any part of the pup’s body, primarily the ano-genital region;
– Sniffing: the dam was sniffing one or more pups;
– Nursing: part of the litter was attached to dam’s nipples while the dam did not show obvious back-arching;
– Crouching (or arched-back nursing): the dam was domed over all pups with the body arched, hind-limbs splayed and no apparent movement;
– Nest Building: the dam was pushing and pulling the sawdust toward the pups to form a nest.

*Non-pup-directed behaviors*:

– Digging: the dam was nuzzling in the sawdust out of the nest area, pushing and kicking it around using the snout and/or both fore- and hind-paws;
– Grooming: the dam was wiping, licking, combing or scratching any part of its own body;
– Wall Rearing: the dam was rearing on hindlimbs, while leaning (or not) with forelimbs on the cage walls, often sniffing the air;
– Exploring: the dam was moving around the cage and sniffing the sawdust, but not carrying pups or nesting material;
– Resting: the dam was lying down alone, out of the nest.

*Other behaviors*: all behaviors different from the ones classified in the previous categories.

Manual scoring was performed by a researcher blind to dams’ rearing conditions by using Ethovision XT (Noldus, The Netherlands). Data analysis was performed on total duration of the previously described behaviors and on sum of *pup-directed, non-pup-directed* and *other behaviors* during 30 min-observation period.

#### Pups

##### Morris water maze

The rats were placed in a circular white pool (diameter 140 cm) filled with 24°C water made opaque by the addition of atoxic acrylic color (Giotto, Italy; Cutuli et al., 2009). An escape platform (diameter 10 cm) was submerged 2 cm below the water level. Each rat was submitted to a 10-trial Place phase followed by a 1-trial Probe phase with an inter-phase interval of 3 min. During Place trials, the rat was released into the water from randomly varied starting points and allowed to find the hidden platform for a maximum of 60 s with an inter-trial interval of 30 s. When the rat reached the platform, it was allowed to remain there for 30 s. If the rat failed to reach the hidden platform within 60 s, it was gently guided there by the experimenter. During Probe trial, the platform was removed and rat was allowed to swim for 30 s in searching for it. Navigational trajectories were recorded by a video camera whose signal was relayed to a monitor and to the previously described image analyzer.

The following MWM parameters were considered: *latencies* to find the platform; *total distance* swum in the pool; mean *swimming velocity*; percentage of *time* spent in the previously rewarded quadrant during Probe phase; *navigational strategies* put into action in reaching the platform. The navigational strategies were classified in two main categories, regardless the platform was reached or not: Searching, swimming around the pool initially with circular trajectories and then exploring the whole pool area; Finding, swimming toward the platform without any foraging around the pool. Two researchers who were unaware of the individual specimen’s group categorized the swimming trajectories drawn by the image analyzer. They attributed the dominant behavior in each trial to a specific category (inter-rate reliability > 0.85).

#### Biochemical Assay

##### Tissue dissection

The animals were decapitated and the brains were quickly removed and dissected on ice by using a binocular dissection microscope. The following brain regions were collected according to Glowinski and Iversen’s (1966) method: frontal cortex and hippocampus of dams and pups at pups’ weaning (pnd 21). All brain regions were extracted in 1 ml extraction buffer/100 mg tissue. Brain tissue samples were homogenized in an ice-cold lysis buffer containing 137 mM NaCl, 20 mM Tris–HCl (pH 8.0), 1% NP40, 10% glycerol, 1 mM phenylmethanesulfonylfluoride (PMSF), 10 mg/ml aprotinin, 1 mg/ml leupeptin, and 0.5 mM sodium vanadate. The tissue homogenate solutions were centrifuged at 14000 × *g* for 25 min at 4°C. The supernatants were collected and stored at –80°C until analyses.

##### BDNF determination by Enzyme-Linked Immunosorbent Assay (ELISA)

Concentrations of BDNF protein were assessed using a two-site enzyme immunoassay kit (Promega, Madison, WI, USA). In brief, 96-well immunoplates (NUNC) were coated with 50 μl/well with the corresponding capture antibody which binds the neurotrophin of interest, and stored overnight at 4°C. The next day serial dilutions of known amounts of BDNF ranging from 0 to 500 pg/ml were performed in duplicate to generate a standard curve. Then the plates were washed three times with wash buffer and the standard curves and supernatants of brain tissue homogenates were incubated in the coated wells (100 μl each) for 2 h at room temperature (RT) with shaking. After additional washes, the antigen was incubated with second specific antibody for 2 h at RT (BDNF), as specified in the protocol. The plates were washed again with wash buffer and then incubated with an anti-IgY HRP for 1 h at RT. After another wash, the plates were incubated with a TMB/Peroxidase substrate solution for 15 min and phosphoric acid 1M (100 μl/well) was added to the wells. The colorimetric reaction product was measured at 450 nm using a microplate reader (Dynatech MR 5000, Germany). BDNF concentrations were determined from the regression line for the neurotrophin standard (ranging from 7.8 to 500 pg/ml-purified mouse BDNF) incubated under similar conditions in each assay. Cross-reactivity with other related neurotrophic factors, for example, NGF, NT-3, and NT-4 was less than 3%. BDNF concentration was expressed as pg/g wet weight and all assays were performed in triplicate.

### Statistical Analyses

Statistical analyses were performed by using STATISTICA 8.0 (StatSoft, Italy). The data expressed as mean ± SEM were firstly tested for normality (Wilk–Shapiro’s test) and homoscedasticity (Levene’s test), and then analyzed by one-way or two-way ANOVAs. The main factor of one-way ANOVAs was the mother pre-reproductive rearing condition. The main factors of two-way ANOVAs were “foster mother”, referring to the rearing condition of adoptive mother, and “biological mother”, referring to the rearing condition of the biological mother of the adopted pups. ANOVAs were followed by HSD Tukey’s test when appropriate. When parametric assumptions were not fully met, non-parametric analyses of variance (Friedman’s test or Kruskal–Wallis’ test followed by Mann–Whitney’s) were used. Differences were considered significant at the *p* < 0.05 level.

## Results

### Behavioral Testing

#### Litter Size, Sex Ratio, and Weight

At end of the EE exposure (pnd 72), EF weighted significantly less than SF [EF: 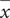 = 211.07 ± 3.64 g; SF: 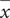 = 241.40 ± 4.58 g; *F*_(1,38)_ = 18.63, *p* = 0.0001].

**FIGURE 2:**
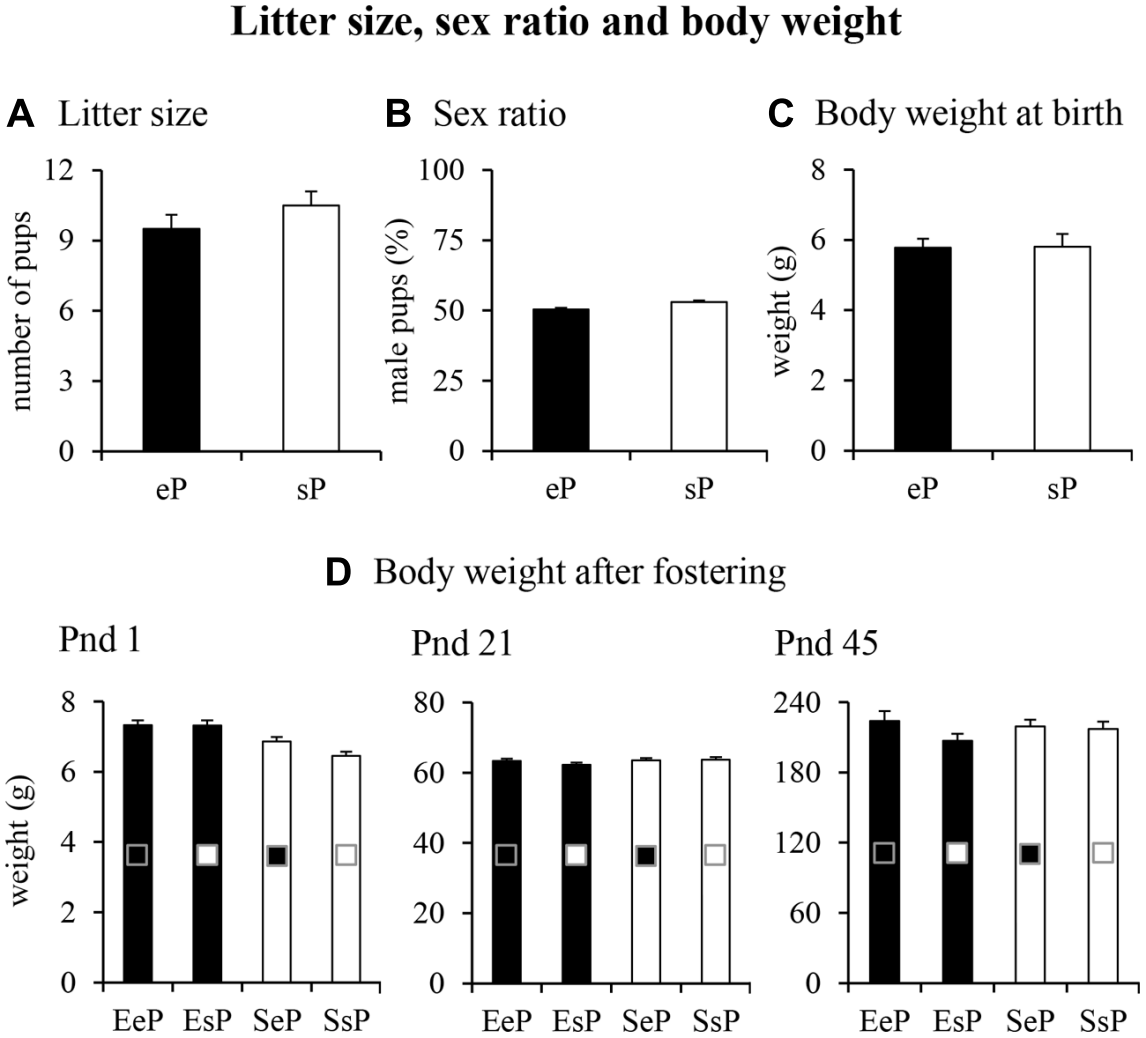
**Results of pre-reproductive maternal enrichment and fostering manipulations on litter size, sex ratio and body weight: histograms show litter size **(A)**, sex ratio **(B)**, and body weight at birth **(C)** of pups from EF (eP) and SF (sP), and the body weight at pnd 1, 21, and 45 **(D)** analyzed in EeP, SsP, EsP, and SeP.** In this and in the following figures, data are reported as mean ± SEM.

With regard to litter characteristics (**Figure 2**), pre-reproductive maternal enrichment did not significantly affect litter size [*F*_(1,22)_ = 1.55, *p* = 0.23] and sex ratio [percentage of male pups: *F*_(1,18)_ = 0.0005, *p* = 0.98]. At birth (pnd 0) the body weight of pups born to EF did not differ from that of pups born to SF [*F*_(1,125)_ = 0.07, *p* = 0.79]. Even after fostering manipulations, the body weight of pups was similar among groups at pnd 1 [two-way ANOVAs: foster mother effect: *F*_(1,123)_ = 3.27, *p* = 0.07; biological mother effect: *F*_(1,123)_ = 2.65, *p* = 0.11; interaction: *F*_(1,123)_ = 3.03, *p* = 0.08], pnd 21 [foster mother effect: *F*_(1,60)_ = 1.46, *p* = 0.23; biological mother effect: *F*_(1,60)_ = 0.58, *p* = 0.45; interaction: *F*_(1,60)_ = 1.01, *p* = 0.32], and pnd 45 [foster mother effect: *F*_(1,44)_ = 0.16, *p* = 0.69; biological mother effect: *F*_(1,44)_ = 2.04, *p* = 0.16; interaction: *F*_(1,44)_ = 1.171, *p* = 0.28; **Figure 2**].

#### Dams

##### Nest building activity

**FIGURE 3:**
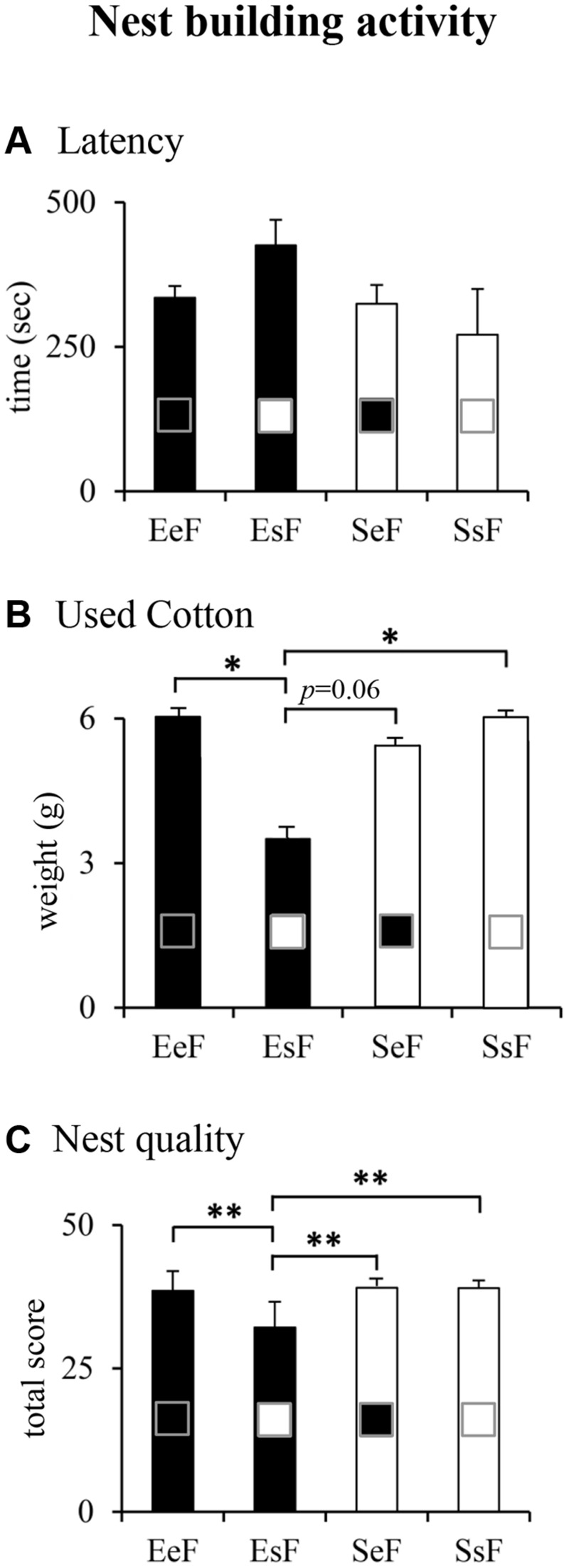
**Results of pre-reproductive maternal enrichment and fostering manipulations on nest building activity: histograms show quantitative (latency, **A**; used cotton, **B**) and qualitative (nest quality, **C**) indexes of nest building activity of EeF, EsF, SeF, and SsF dams (^∗^*p* < 0.05, ^∗∗^*p* < 0.01)**.

Kruskal–Wallis’s test followed by Mann–Whitney’s *U* performed on nest building indexes revealed that dams did not differ in the latency to manipulate cotton (**Figure 3A**). However, EsF (EF rearing pups born to a SF) used less cotton and obtained the lowest score for nest building in comparison to the other dams (**Figures 3B,C**). The results of non-parametric analyses are reported in Supplementary Table S1.

In summary, EsF dams were engaged in building nests of the lowest shape complexity.

##### Maternal care observations

**FIGURE 4:**
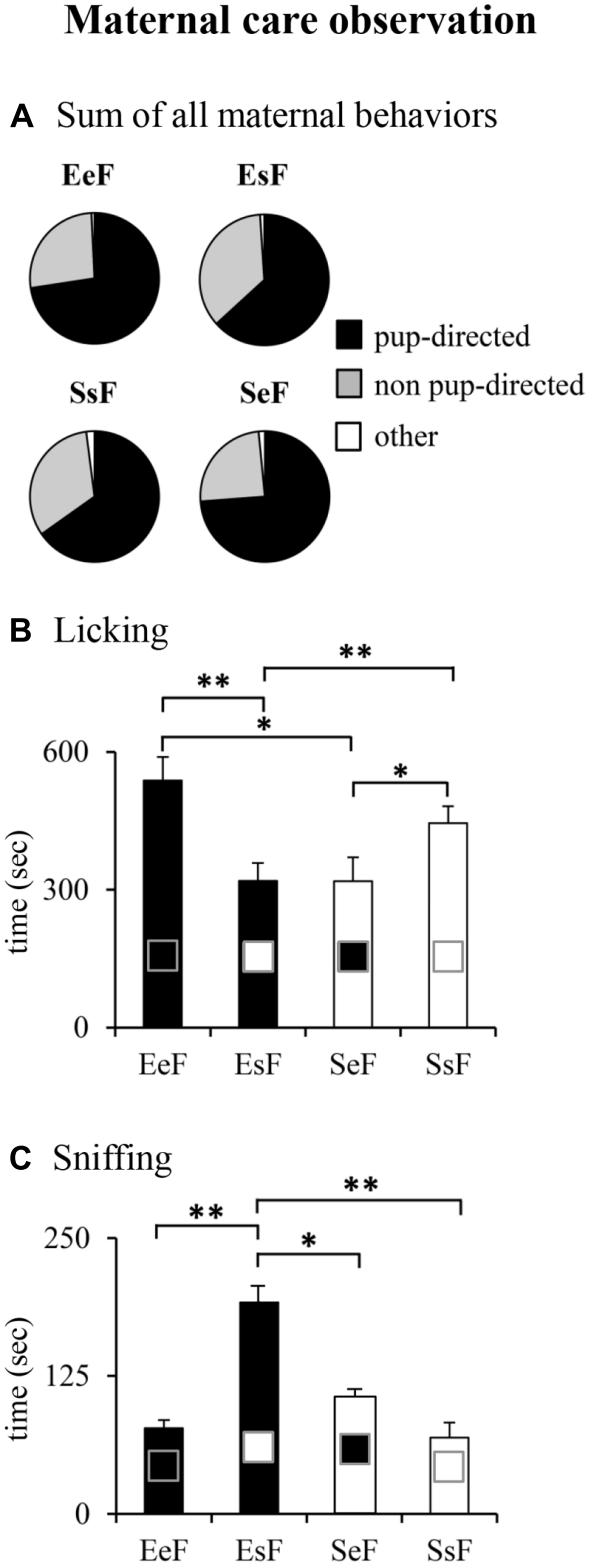
**Results of pre-reproductive maternal enrichment and fostering manipulations on maternal behaviors: pie charts show sum of different kind of maternal behaviors (pup-directed, non-pup-directed and other behaviors) **(A)**.** Histograms show total duration of Licking **(B)**, and Sniffing **(C)** behaviors of EeF, EsF, SeF, and SsF dams (^∗^*p <* 0.05, ^∗∗^*p* < 0.01).

Typically, when the dams received their pups after the 30-min isolation period, all of them quickly approached the litter and sniffed the pups. Then they retrieved the litter and began to manipulate the sawdust to create a nest area. After brief explorations of the home cage as the observation went by they began to lick and nurse pups. The temporal evolution of maternal care was not influenced by the enrichment and fostering manipulations, as shown in Supplementary Figure S1 depicting the single *pup-directed* and *non-pup-directed behaviors* displayed in three 10-min blocks of observation. Kruskal–Wallis’s test performed on the sums of *pup-directed, non-pup-directed* and *other behaviors* of the entire 30 min-observation revealed no differences among dams (**Figure 4A**). Detailed analyses performed on the single *pup-directed behaviors* demonstrated that both SeF and EsF (*cross-fostered* groups) showed lower *Licking* duration (**Figure 4B**) in comparison to SsF and EeF (*in-fostered* groups). Moreover, EsF showed the longest *Sniffing* duration (**Figure 4C**). No differences were observed in the remaining behaviors. The results of non-parametric analyses are reported in Supplementary Table S2.

In summary, at ppd 1 the dams reared in a given condition and rearing pups born to a female exposed to a different rearing condition (*cross-fostered* groups) were less engaged in maternal care.

#### Offspring

##### Morris water maze

**FIGURE 5:**
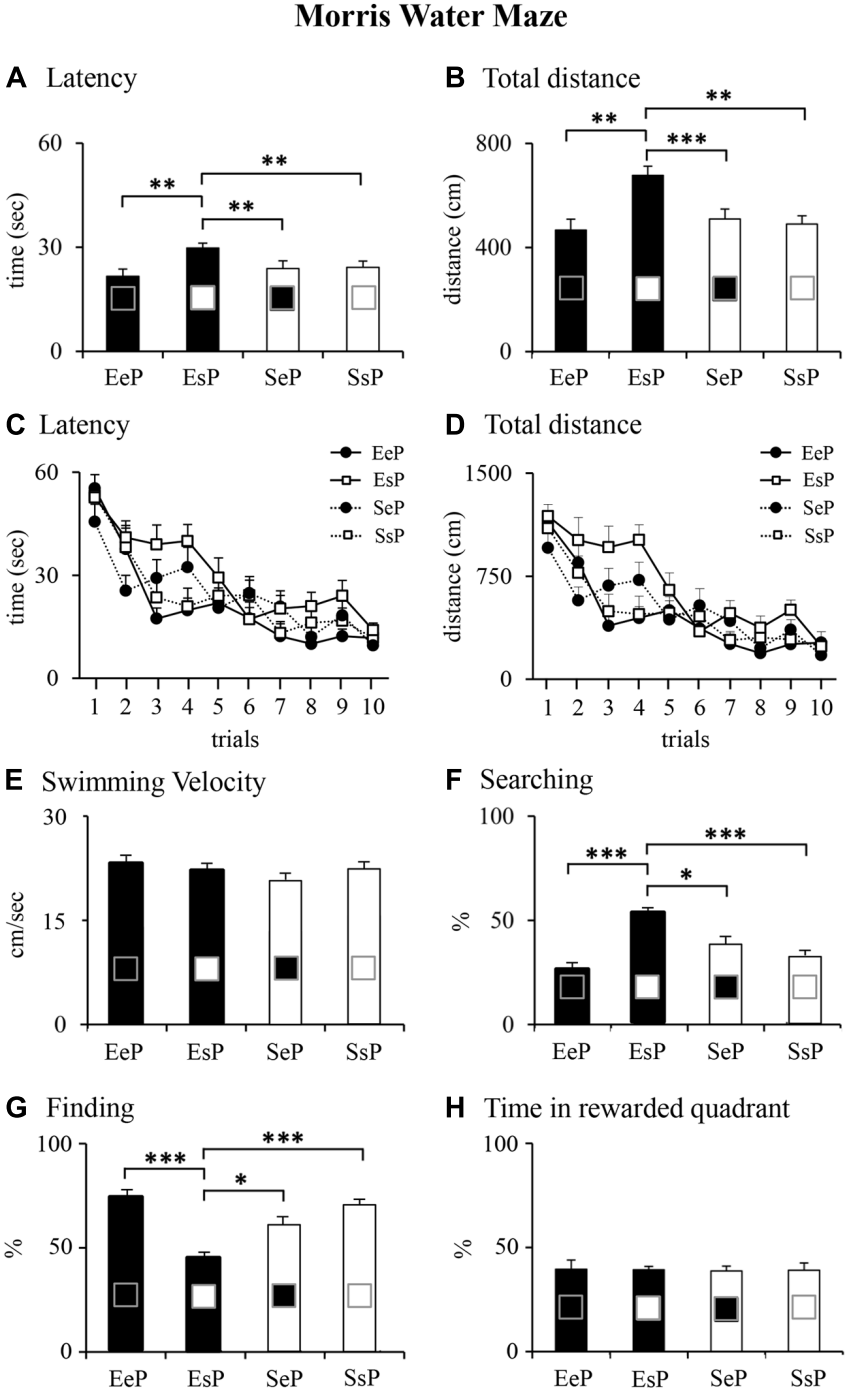
**Results of pre-reproductive maternal enrichment and fostering manipulations on offspring spatial performances: histograms show latency (**A,C**), total distance (**B,D**) swimming velocity **(E)**, and navigational strategies put into action (Searching, **F**; Finding, **G**) throughout MWM test, and percentage of time spent in the previously rewarded quadrant during Probe phase **(H)** of EeP, SsP, EsP, and SeP rats (^∗^*p* < 0.05, ^∗∗^*p* < 0.01, ^∗∗∗^*p* < 0.001)**.

Animals reared by an enriched female and born to a standard female (EsP) employed significantly more time and traveled longer distances to reach the hidden platform in comparison to other rats, as revealed by Kruskal–Wallis’s tests followed by Mann-Whitney’s U tests performed on *latency* and *total distance* (**Figures 5A,B**). Friedman analyses on *latency* and *total distance* revealed that all groups learned to reach the platform as trials went by (**Figures 5C,D**). No difference among groups was found on *swimming velocity* (**Figure 5E**). As for *navigational strategies*, EsP rats showed the highest percentage of Searching and the lowest percentage of Finding (**Figures 5F,G**). Finally, Kruskal–Wallis’s test performed on percentage of time spent in searching the platform in the rewarded quadrant during the Probe phase failed to reveal significant differences among groups (**Figure 5H**). These statistical results are reported in Supplementary Table S3.

In summary, all groups learned to reach the platform in the Place phase although the standard offspring cross-fostered by an enriched dam (EsP) displayed the poorest spatial learning performance. During the Probe phase all groups remembered the platform localization, indicating intact spatial memory.

### BDNF Levels

#### Dams

**FIGURE 6:**
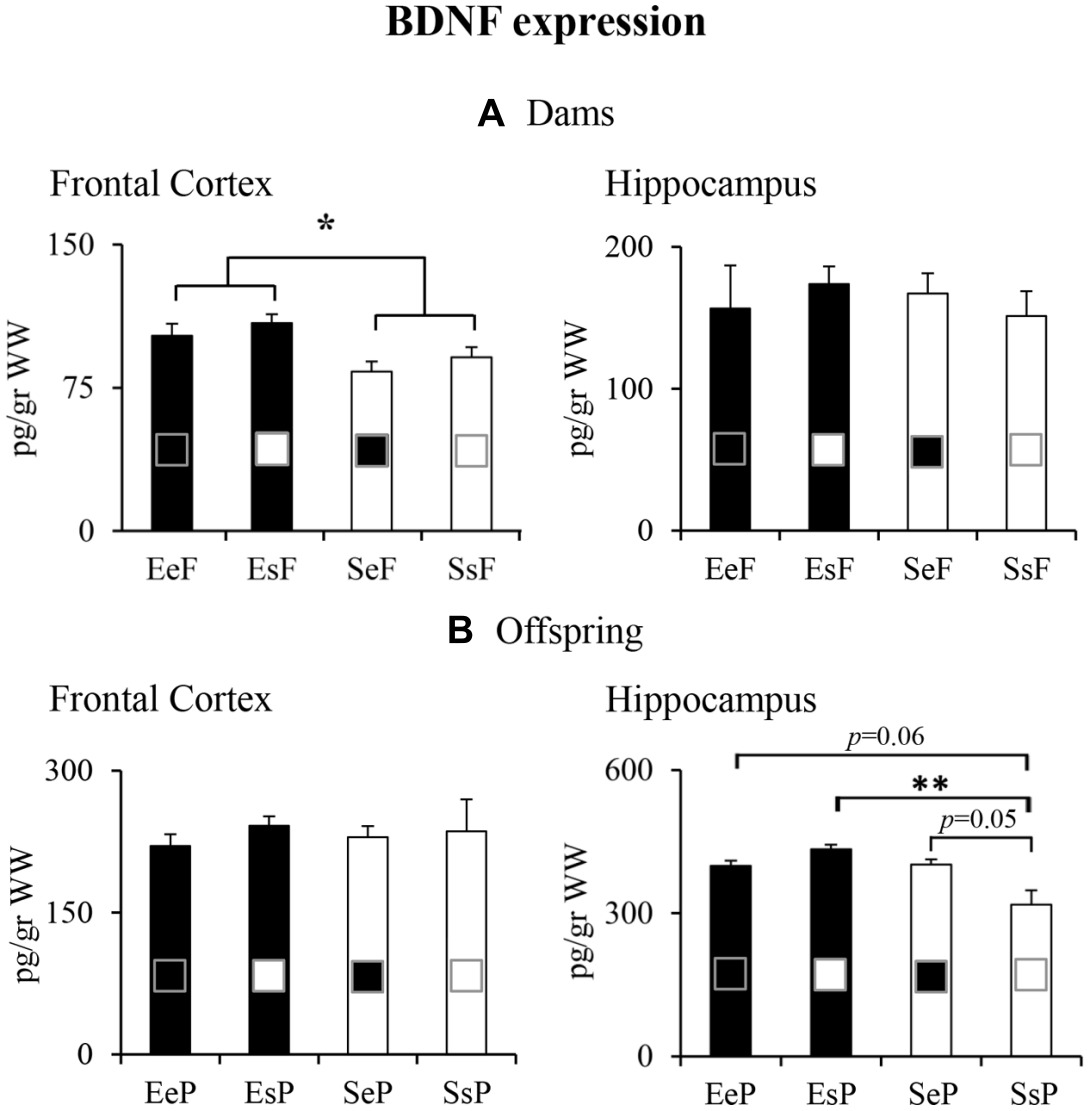
**Results of pre-reproductive maternal enrichment and fostering manipulations on BDNF expression: histograms show BDNF expression levels analyzed in frontal cortex and hippocampus of EeF, SsF, EsF, and SeF dams **(A)**, and of EeP, SsP, EsP, and SeP offspring **(B)** (^∗^*p* < 0.05, ^∗∗^*p* < 0.01)**.

Regardless of the kind of pups received, the EF had frontal BDNF levels significantly higher than the SF [two-way ANOVA: foster mother effect: *F*_(1,12)_ = 6.94, *p* = 0.02; biological mother effect: *F*_(1,12)_ = 1.07, *p* = 0.32; interaction: *F*_(1,12)__=_ 0.004, *p* = 0.95]. No differences among groups were found analyzing BDNF expression levels in the hippocampus [two-way ANOVA: foster mother effect: *F*_(1,12)_ = 0.08, *p* = 0.78; biological mother effect: *F*_(1,12)_ = 0.001, *p* = 0.97; interaction: *F*_(1,12)__=_ 0.59, *p* = 0.46; **Figure 6A**].

In summary, the pre-reproductive EE exposure induced a long-lasting enhancement of frontal BDNF expression in dams, while fostering manipulations did not perturb neurotrophin expression levels.

#### Offspring

Pups reared by and born to a standard female (SsP) had the lowest hippocampal BDNF levels in comparisons to the remaining groups (EeP, EsP, and SeP). In particular, while EeP and SeP just tended, EsP significantly show increased BDNF levels in comparison to SsP [two-way ANOVA: foster mother effect: *F*_(1,12)_ = 7.85, *p* = 0.02; biological mother effect: *F*_(1,12)_ = 1.44, *p* = 0.25; interaction: *F*_(1,12)_ = 8.62, *p* = 0.01]. No differences among groups were found analyzing BDNF expression levels in the frontal cortex [two-way ANOVA: foster mother effect: *F*_(1,12)_ = 0.007, *p* = 0.94; biological mother effect: *F*_(1,12)_ = 0.41, *p* = 0.53; interaction: *F*_(1,12)__=_ 0.12, *p* = 0.73; **Figure 6B**].

In summary, pups born to and/or reared by an enriched female tended to show greater hippocampal BDNF expression.

## Discussion

In recent studies, we demonstrated that pre-reproductive maternal EE exerts beneficial effects on maternal behaviors as well as on motor development and spatial cognition of the offspring, and enhances the brain neurotrophin expression in both dams and pups (Caporali et al., 2014; Cutuli et al., 2015). However, since the mother represents the primary link between environment and pup, and even subtle variations in maternal care have a profound impact on offspring development, these studies did not allow evaluating whether the transgenerational beneficial effects of EE were a product of prenatal factors, post-natal experience or both. Therefore, to disentangle the effects of maternal nurturance from those of genetic transmission, we performed the present cross-fostering study, using in-fostered groups as controls for the adoption effects.

Unexpectedly, the pre-reproductive maternal EE followed by adoption manipulations no more exerted beneficial effects on maternal care and offspring performance (as in Cutuli et al., 2015). In fact, the enriched dams rearing in-fostered pups (EeF) did not show any difference in maternal care in comparison to standard dams rearing in-fostered pups (SsF), indicating that the adoption procedure *per se* exerted a detrimental effect on enriched females’ maternal behavior. In addition, the dams of both cross-fostered groups (EsF and SeF) were less engaged in licking their pups in comparison to the dams of both in-fostered groups (EeF and SsF). Even, the enriched dams that cross-fostered standard pups (EsF) showed the least complex maternal care repertoire, exhibiting the lowest nest quality and highest sniffing duration. Notably, the olfaction is a sensory modality of singular importance for the fine adjustment of early mother-infant interactions, with the olfactory cues involved in various aspects of maternal care. Given females commonly sniff at pup’s head and body regions in which the skin glands are located, infantile odors are very potent stimuli that provide the basis for maternal recognition of the pups and so allow the normal engagement of maternal nurturing (Lévy and Keller, 2009). The olfactory “signature” of a pup reflects in fact the interaction between its genotype and the early (intra- and extra-uterine) environment which it is exposed to (Lévy et al., 2004). The long-lasting olfactory exploration of EsF dams may be due to a “doubtful” recognition of pup’s olfactory signatures by the mother (“is it really my pup?”) but also to different signaling through sight, motor activity, and vocalization provided by the pup (“is it really my mum?”). In this regard, pups’ distal sensory stimuli (sight, sound, and odor) trigger maternal contact-seeking behaviors, whereas pups’ proximal tactile stimuli (touching dam’s snout and body) elicit the maternal retrieval, licking, and nursing of pups (Stern, 1997). Indeed, it is well known that mothers modify the type of care they provide to the litter in response to the requirements of the offspring itself (Moore, 1982; Stern and Johnson, 1990). Interestingly, lactating dams enhance their maternal behavior in the presence of highly demanding pups (Pereira and Ferreira, 2006), supporting the idea that maternal behavior is, at least partially, a response to the motivational cues from pups. In this framework, we advance that standard pups could not adequately (too much or too little?) stimulate their adoptive enriched dams which thus showed a worse maternal behavior, as demonstrated by the lowest nest building quality score. Then, the poor maternal care exhibited by EsF dams could reflect alteration in bidirectional interaction with cross-fostered standard pups. Future studies will clarify the specific load of the maternal and filial components in this maladaptive interaction. Not by chance, the limited maternal investment of EsF went along with the reduced spatial learning performances of their cross-fostered offspring (EsP) in the MWM test. In fact, during the Place phase EsP reached the platform through the scarcely efficient Searching strategy showing a delayed spatial learning, even if in the Probe phase remembered the platform localization as the remaining groups. The close relationship between maternal behavior and offspring performances fits with previous findings demonstrating that low maternal care impairs hippocampal development and spatial functions of the offspring (Meaney, 2001). Namely, offspring of mothers that exhibit low levels of licking/grooming and arched-back nursing (low LG-ABN mothers) shows compromised spatial learning in comparison to animals reared by high LG-ABN mothers (Francis and Meaney, 1999; Bredy et al., 2004). Altered offspring’s MWM performances have been described also following maternal separation (Cao et al., 2014). Furthermore, the observation that MWM performances of EeP and SsP groups were very similar once more indicates that maternal EE in the presence of adoption procedures did not provide the beneficial influences previously reported in Cutuli et al. (2015) on offspring spatial performances. Conversely, the biochemical analyses on BDNF expression of the dams revealed that the adoption procedures did not negatively affect this neuroplastic correlate. In fact, regardless of the pups received, the cross- and in-foster enriched females exhibited high BDNF levels in the frontal cortex, structure involved in modulating maternal care in rodents (Afonso et al., 2007; Febo and Ferris, 2007; Febo et al., 2010) as well as in humans (Lorberbaum et al., 2002; Ranote et al., 2004; Swain, 2008). This finding is in line with data showing enhanced BDNF levels in the frontal cortex of pre-reproductively enriched dams (Cutuli et al., 2015). However, in that study we could not ascertain whether in the enriched dams the enhanced BDNF expression was linked to the exposure to pre-reproductive EE or resulted by their increased maternal care, or both of them. In the present study, the lacking differences in maternal care between in-fostered groups (EeF and SsF), even in the presence of increased BDNF levels in both groups of enriched dams, suggest that the increase in frontal neurotrophin levels is linked to pre-reproductive EE exposure.

As for the offspring, cross- and in-fostered pups born to enriched dams (SeP and EeP) tended to exhibit higher hippocampal BDNF expression in comparison to standard pups (SsP). This finding suggests that the pre-reproductive maternal EE previously seen to enhance pups’ neurotrophin expression (Cutuli et al., 2015) when combined to adoption procedure no more resulted in an overt BDNF increase. Differently, a significant increase in BDNF expression was found in the standard pups reared by enriched dams (EsP). Notably, the increased hippocampal neurotrophin expression was found just in the pups reared by the mothers showing the worst maternal care (EsF). Hippocampal BDNF levels are highly sensitive to parental experiences (Arai et al., 2009; Roth et al., 2009; Mychasiuk et al., 2012) and complex environmental stimulations (Pham et al., 2002; Gelfo et al., 2011; Chourbaji et al., 2012). Interestingly, Suri et al. (2013) and Nishinaka et al. (2015) demonstrated that animals subjected to early stress of maternal separation exhibited enhanced hippocampal BDNF levels from weaning until young adulthood. Thus, maternal separation as well as early adverse mother-pup interactions may transiently result in increased expression of hippocampal neurotrophin.

In the present study, offspring biochemical data did not fully correlate with spatial performances. Namely, EsP showed high BDNF levels and reduced spatial learning (as indicated by their enhanced use of an indirect navigational strategy), but an intact spatial memory (as indicated by time spent in the previously rewarded quadrant). The literature supports a role for hippocampal BDNF expression in both learning acquisition and consolidation (Tyler et al., 2002; Leal et al., 2015). However, numerous findings suggest that the link between BDNF and learning and memory is not as direct as expected (Fischer et al., 1994; Pelleymounter et al., 1996; Montkowski and Holsboer, 1997; Croll et al., 1999; Cirulli et al., 2000). A speculative interpretation to explain EsP results can take into account the relevance of BDNF-TrkB signaling on hippocampal-dependent learning (Tyler et al., 2002). It has been reported that *knockout* mice with altered expression of the BDNF receptor TrkB exhibit impairment in hippocampal-mediated learning (Minichiello et al., 1999; Saarelainen et al., 2000). In this framework, we can hypothesize that the high BDNF expression of EsP could go along with an impaired functioning of TrkB receptors (in line with their reduced spatial learning), and then the BDNF increase could be the outcome of a compensatory mechanism in BDNF-TrkB signaling. Further studies have to be performed to clarify this issue.

In summary, the present findings enlighten the crucial importance of the early mother-pups interactions in influencing maternal care and offspring phenotype, with the enriched dam-standard pups couple resulting in the most maladaptive encounter. The feedback loop between mother and offspring is of potential importance for understanding the causes of many developmental outcomes. Changes in maternal care have salient effects on the offspring, including epigenetic alterations that affect the transmission of traits across generations (Champagne and Curley, 2009). On the other hand, changes in maternal behavior can be due to changes in the behavior or altered signals transmitted by the offspring (Cummings et al., 2010).

## Conclusion

It is necessary to acknowledge that any environmental factor not only impinges on the mother and offspring, but also on their interactions and social bond. Just the mutual interactions between mother and pups may trigger epigenetic mechanisms active in shaping offspring phenotype.

## Author Contributions

LP, DC, and FA designed the research; DC, PC, DL, and FF performed behavioral evaluation; FG, PB, and FA performed biochemical analyses; PC, DC, FG, and LP analyzed data; all authors discussed and approved data; PC, DC, DL, and LP wrote the paper.

## Conflict of Interest Statement

The authors declare that the research was conducted in the absence of any commercial or financial relationships that could be construed as a potential conflict of interest.
