# Supplementary material for: Interaction does Count: A Cross-Fostering Study on Transgenerational Effects of Pre-reproductive Maternal Enrichment
Source: Front Behav Neurosci. 2015 Dec 1;9:320. doi: 10.3389/fnbeh.2015.00320 (PMC4665747; doi:10.3389/fnbeh.2015.00320)
Supplement: Supplementary file 1 [file Table_1.DOCX]

|  | | Latency to manipulate cotton | Used cotton  weight | Nest quality  score |
| --- | --- | --- | --- | --- |
| Kruskal-Wallis’s test | | H=3.93;  *p*=0.27 | **H=10.24; *p*=0.02** | **H=11.67; *p*=0.009** |
| Mann-Whitney’s test | EeF *vs.* SsF |  | Z=0.01;  *p*=1 | Z=0.81;  *p*=0.49 |
|  | EeF *vs.* EsF |  | **Z=2.29;**  ***p*=0.02** | **Z=2.58;**  ***p*=0.009** |
|  | SsF *vs.* SeF |  | Z=1;  *p*=0.70 | Z=0.73;  *p*=0.49 |
|  | EeF *vs.* SeF |  | Z=1;  *p*=0.70 | Z=-0.33;  *p*=0.82 |
|  | SsF *vs.* EsF |  | **Z=2.29;**  ***p*=0.02** | **Z=2.58;**  ***p*=0.009** |
|  | EsF *vs.* SeF |  | Z=1.91;  *p*=0.06 | **Z=2.90;**  ***p*=0.002** |

**Supplementary Table 1. Nest building activity.** Statistical significance of data comparisons is reported.
