# Supplementary material for: Interaction does Count: A Cross-Fostering Study on Transgenerational Effects of Pre-reproductive Maternal Enrichment
Source: Front Behav Neurosci. 2015 Dec 1;9:320. doi: 10.3389/fnbeh.2015.00320 (PMC4665747; doi:10.3389/fnbeh.2015.00320)
Supplement: Supplementary file 2 [file Table_2.DOCX]

| **Pup-directed**  **behaviors** | | Sum | Retrieving | Licking | Sniffing | Nursing | Crouching | Nest building |
| --- | --- | --- | --- | --- | --- | --- | --- | --- |
| Kruskal-Wallis’s test | | H=2.47; *p*=0.48 | H=1.92; *p*=0.59 | **H=11.78; *p*=0.008** | **H=13.89; *p*=0.003** | H=0.47; *p*=0.98 | H=2.15; *p*=0.54 | H=2.81; *p*=0.42 |
| Mann-Whiney’s tests | EeF *vs.* SsF |  |  | Z=0.96;  *p*=0.39 | Z=-0.16;  *p*=0.94 |  |  |  |
|  | EeF *vs.* EsF |  |  | **Z=2.56;**  ***p*=0.009** | **Z=-2.88;**  ***p*=0.008** |  |  |  |
|  | SsF *vs.* SeF |  |  | **Z=2.08;**  ***p*=0.04** | Z=-1.92;  *p*=0.06 |  |  |  |
|  | EeF *vs.* SeF |  |  | **Z=2.24;**  ***p*=0.03** | Z=-1.60;  *p*=0.13 |  |  |  |
|  | SsF *vs.* EsF |  |  | **Z=2.40;**  ***p*=0.01** | **Z=-2.88;**  ***p*=0.008** |  |  |  |
|  | EsF *vs.* SeF |  |  | Z=-0.64;  *p*=0.59 | **Z=2.08;**  ***p*=0.04** |  |  |  |
|  | | | | | | | | |
| **Non pup-directed**  **behaviors** | | Sum | Digging | Grooming | Wall rearing | Exploring | Resting | **Other**  **behaviors** |
| Kruskal-Wallis’s test | | H=1.62; *p*=0.65 | H=6.15; *p*=0.10 | H=6.59; *p*=0.08 | H=3.56; *p*=0.21 | H=3.33; *p*=0.34 | H=7.74; *p*=0.51 | H=3.81; *p*=0.22 |

**Supplementary Table 2. Maternal behavior observation:** Statistical significance of data comparisons is reported.
