# Supplementary material for: Interaction does Count: A Cross-Fostering Study on Transgenerational Effects of Pre-reproductive Maternal Enrichment
Source: Front Behav Neurosci. 2015 Dec 1;9:320. doi: 10.3389/fnbeh.2015.00320 (PMC4665747; doi:10.3389/fnbeh.2015.00320)
Supplement: Supplementary file 3 [file Table_3.DOCX]

|  | | Latency | Total  distance | Swimming  velocity | Navigational strategies | | Probe |
| --- | --- | --- | --- | --- | --- | --- | --- |
|  |  |  |  |  | Searching | Finding |  |
| Kruskal-Wallis’s test | | **H=11.47; *p*=0.009** | **H=14.58; *p*=0.002** | H=1.44;  *p*=0.70 | **H=18.78; *p*=0.0003** | **H=18.46; *p*=0.0004** | H=0.56;  *p*=0.91 |
| Mann-Whitney’s test | EeP *vs.* SsP | Z=-1.31;  *p*=0.19 | Z=-1.29;  *p*=0.21 |  | Z=-1.29;  *p*=0.21 | Z=1.08;  *p*=0.30 |  |
|  | EeP *vs.* EsP | **Z=-2.89;**  ***p*=0.003** | **Z=-3.39;**  ***p*=0.0005** |  | **Z=-3.39;**  ***p*=0.0005** | **Z=3.42;**  ***p*=0.0005** |  |
|  | SsP *vs.* SeP | Z=0.10;  *p*=0.94 | Z=-1.38;  *p*=0.19 |  | Z=-1.38;  *p*=0.19 | Z=1.38;  *p*=0.19 |  |
|  | EeP *vs.* SeP | Z=-1.10;  *p*=0.29 | Z=-1.96;  *p*=0.05 |  | Z=-1.96;  *p*=0.05 | Z=1.87;  *p*=0.07 |  |
|  | SsP *vs.* EsP | **Z=-2.49;**  ***p*=0.01** | **Z=-3.80;**  ***p*=0.00004** |  | **Z=-3.80;**  ***p*=0.00004** | **Z=3.80;**  ***p*=0.00004** |  |
|  | EsP *vs.* SeP | **Z=-2.43;**  ***p*=0.01** | **Z=2.26;**  ***p*=0.03** |  | **Z=2.26;**  ***p*=0.03** | **Z=-2.26;**  ***p*=0.03** |  |
|  | | Latency | Total  distance |  |  |  |  |
| Friedman’s test | EeP | **x^2^=45.33;**  ***p*<0.000001** | **x^2^=44.20;**  ***p*<0.000001** |  |  |  |  |
|  | EsP | **x^2^=53.97;**  ***p*<0.000001** | **x^2^=57.87;**  ***p*<0.000001** |  |  |  |  |
|  | SeP | **x^2^=43.39;**  ***p*<0.000001** | **x^2^=43.51;**  ***p*<0.000001** |  |  |  |  |
|  | SsP | **x^2^=40.49;**  ***p*=0.00001** | **x^2^=35.94;**  ***p*=0.00004** |  |  |  |  |

**Supplementary Table 3. Morris water Maze.** Statistical significance of data comparisons is reported.
