# Supplementary figures and images for: Interaction does Count: A Cross-Fostering Study on Transgenerational Effects of Pre-reproductive Maternal Enrichment
Source: Front Behav Neurosci. 2015 Dec 1;9:320. doi: 10.3389/fnbeh.2015.00320 (PMC4665747; doi:10.3389/fnbeh.2015.00320)

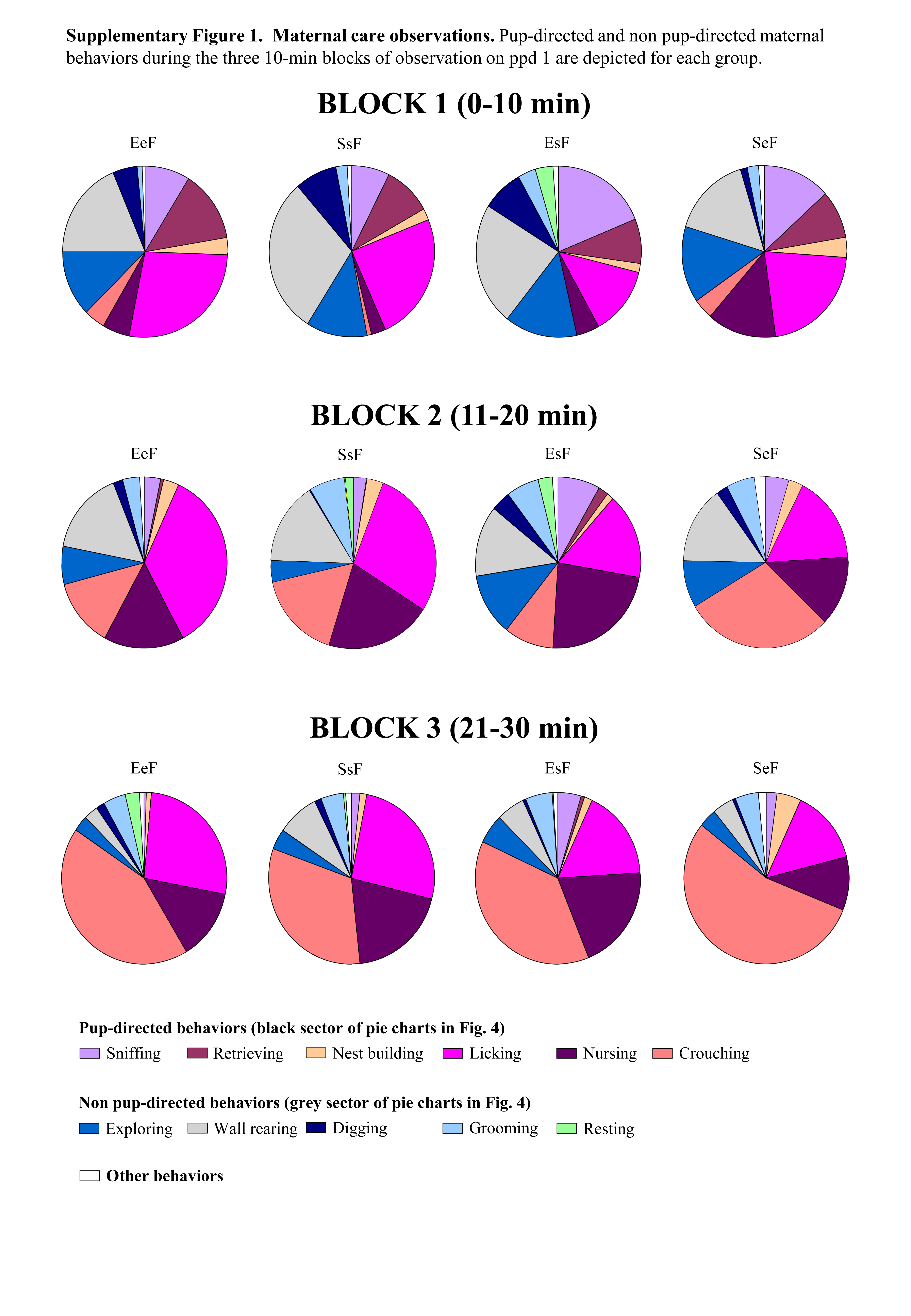

Supplement: Supplementary file 4 [file Image_1.TIF]
